# OfWRKY17-OfC3H49 module responding to high ambient temperature delays flowering via inhibiting *OfSOC1B* expression in *Osmanthus fragrans*

**DOI:** 10.1093/hr/uhae273

**Published:** 2024-09-24

**Authors:** Yong Ye, Xinke Lu, En Kong, Qianqian Wang, Lixiao Shen, Shiwei Zhong, Yiguang Wang, Zheng Xiao, Jinping Deng, Hongbo Zhao, Bin Dong

**Affiliations:** School of Landscape Architecture, Zhejiang Agriculture and Forestry University, No. 666 Wusu Street, Lin'an District, Hangzhou 311300, Zhejiang, China; School of Landscape Architecture, Zhejiang Agriculture and Forestry University, No. 666 Wusu Street, Lin'an District, Hangzhou 311300, Zhejiang, China; School of Landscape Architecture, Zhejiang Agriculture and Forestry University, No. 666 Wusu Street, Lin'an District, Hangzhou 311300, Zhejiang, China; School of Landscape Architecture, Zhejiang Agriculture and Forestry University, No. 666 Wusu Street, Lin'an District, Hangzhou 311300, Zhejiang, China; School of Landscape Architecture, Zhejiang Agriculture and Forestry University, No. 666 Wusu Street, Lin'an District, Hangzhou 311300, Zhejiang, China; School of Landscape Architecture, Zhejiang Agriculture and Forestry University, No. 666 Wusu Street, Lin'an District, Hangzhou 311300, Zhejiang, China; Zhejiang Provincial Key Laboratory of Germplasm Innovation and Utilization for Garden Plants, Zhejiang Agriculture and Forestry University, No. 666 Wusu Street, Lin'an District, Hangzhou 311300, Zhejiang, China; School of Landscape Architecture, Zhejiang Agriculture and Forestry University, No. 666 Wusu Street, Lin'an District, Hangzhou 311300, Zhejiang, China; Zhejiang Provincial Key Laboratory of Germplasm Innovation and Utilization for Garden Plants, Zhejiang Agriculture and Forestry University, No. 666 Wusu Street, Lin'an District, Hangzhou 311300, Zhejiang, China; School of Landscape Architecture, Zhejiang Agriculture and Forestry University, No. 666 Wusu Street, Lin'an District, Hangzhou 311300, Zhejiang, China; Zhejiang Provincial Key Laboratory of Germplasm Innovation and Utilization for Garden Plants, Zhejiang Agriculture and Forestry University, No. 666 Wusu Street, Lin'an District, Hangzhou 311300, Zhejiang, China; School of Landscape Architecture, Zhejiang Agriculture and Forestry University, No. 666 Wusu Street, Lin'an District, Hangzhou 311300, Zhejiang, China; Zhejiang Provincial Key Laboratory of Germplasm Innovation and Utilization for Garden Plants, Zhejiang Agriculture and Forestry University, No. 666 Wusu Street, Lin'an District, Hangzhou 311300, Zhejiang, China; School of Landscape Architecture, Zhejiang Agriculture and Forestry University, No. 666 Wusu Street, Lin'an District, Hangzhou 311300, Zhejiang, China; Zhejiang Provincial Key Laboratory of Germplasm Innovation and Utilization for Garden Plants, Zhejiang Agriculture and Forestry University, No. 666 Wusu Street, Lin'an District, Hangzhou 311300, Zhejiang, China; School of Landscape Architecture, Zhejiang Agriculture and Forestry University, No. 666 Wusu Street, Lin'an District, Hangzhou 311300, Zhejiang, China; Zhejiang Provincial Key Laboratory of Germplasm Innovation and Utilization for Garden Plants, Zhejiang Agriculture and Forestry University, No. 666 Wusu Street, Lin'an District, Hangzhou 311300, Zhejiang, China

## Abstract

Ambient temperature is a pivotal factor in the regulation of the flowering process in plants. In this study, we found that high ambient temperature exerts an inhibitory effect on the flowering of *Osmanthus fragrans* “Sjigui”. However, the underlying molecular mechanisms remain not fully understood. Through transcriptome analysis, a differently expressed C3H gene *OfC3H49* was identified, which is induced by high ambient temperature. OfC3H49 was demonstrated to delay the flowering process of *Arabidopsis* and downregulate the expression of flowering-related genes in *O. fragrans* calli. Further investigation indicates that OfC3H49 as a transcriptional repressor directly suppresses the expression of the *OfSOC1B* thereby causing a delay in flowering time. Furthermore, a WRKY transcription factor, OfWRKY17, was identified to be responsive to high ambient temperature, directly binding to the *OfC3H49* promoter and enhance *OfC3H49* expression. Overexpression of *OfWRKY17* in *Arabidopsis* resulted in a significant delay in flowering and induced the expression of *OfC3H49* in *O. fragrans* calli. Collectively, our findings delineate a regulatory module, OfWRKY17-OfC3H49, which is activated by high ambient temperature and functions as a negative regulator of flowering by suppressing the expression of *OfSOC1B* in *O. fragrans*. This study provides novel insights into the molecular mechanisms involved in ambient temperature-mediated flowering control and contributes to the development of molecular breeding strategies for *O. fragrans*.

## Introduction

Flowering is a critical developmental milestone in the life cycle of plants, marking the transition from vegetive to reproductive growth and determining the success of plant reproduction [1, 2]. The regulatory network that governs flowering has been extensively studied in *Arabidopsis thaliana*, multiple flowering signals ultimately converge upon the key integrators such as *FLOWERING LOCUS T* (*FT*) and *SUPPRESSOR OF OVEREXPRESSION OF CONSTANS 1* (*SOC1*) [3, 4]. Then, these key integrators activate a cascade of floral meristem genes ensuring the flowering occurs in the shoot apical meristem [5].

Ambient temperature serves as a crucial environmental cue for adjusting the floral transition [6]. The sensitivity to temperature cues can vary significantly among species and genetic lines, attributed to their distinct native environments and evolutionary adaptations [7]. In *A. thaliana*, the increase of ambient temperature from 23°C to 27°C significantly induces the flowering by promote the *FT* expression under short-day conditions [8]. Analogous flowering behaviors have also been found in soybean (*Glycine max*) [9] and *Narcissus tazetta* [10], underscoring the important role of high ambient temperature in the flowering regulation. In contrast, a body of research indicates elevated ambient temperatures can exert inhibitory effects on the floral transition in certain plant species [11]. For instance, in *Boechera stricta*, a perennial relative of *Arabidopsis*, the floral initiation is markedly delayed at 25°C as compared to 18°C, suggesting high ambient temperature sensitivity in the inhibition of flowering [12]. Similar temperature-responsive effects on the modulation of flowering have been observed in other species such as Chrysanthemum (*Chrysanthemum morifolium*) [13], *Brassica rapa* [14], and citrus (*Citrus reticulata*) [15]. Nevertheless, the understanding of the molecular mechanism that regulate ambient temperature-responsive flowering in ornamental species, such as *O. fragrans*, is still limited. It is important to elucidate the molecular mechanism underlying the ambient temperature-sensitivity of the flowering regulation in *O. fragrans* and other ornamental plants.

Cysteine3Histidine (CCCH) transcription factors are characterized by a zinc finger motif consisting of 1 to 6 conserved C3H domains (C-X_6–14-_C-X_4–5_-C-X_3_-H) [16]. The CCCH family can be divided into two categories: C3H-tandem ZFPs (TZFs), which contain two C3H-tandem motifs, and non-TZFs, which possess a variable number of C3H motifs [17]. Compared to other types of zinc finger proteins, CCCH zinc fingers are relatively rare, comprising ~0.8% of the zinc finger proteins [18], and they are involved in various biological processes such as leaf senescence [19], secondary wall synthesis [20], and pollen development [21]. Furthermore, evidence indicates that CCCH transcription factors involved in the regulation of flowering in plants. For example, overexpression of the alfalfa (*Medicago sativa*) *C3H* gene *MsZFN* in *Arabidopsis* promotes flowering by upregulating *AtFT* and downregulating *AtFLC* expression [ 22]. In *Adonis amurensis*, the C3H protein AaZFP3 acts as a positive regulator of flowering in transgenic *Arabidopsis* by inducing the transcriptional level of flowering-related genes [23]. CpC3H3, a typical TZF from wintersweet (*Chimonanthus praecox*), is implicated in the induction of flowering by upregulating the expression of genes associated with flowering [24]. However, there is limited report how C3H transcription factors regulate flowering in response to ambient temperature changes in *Arabidopsis* or woody plants.

**Figure 1:**
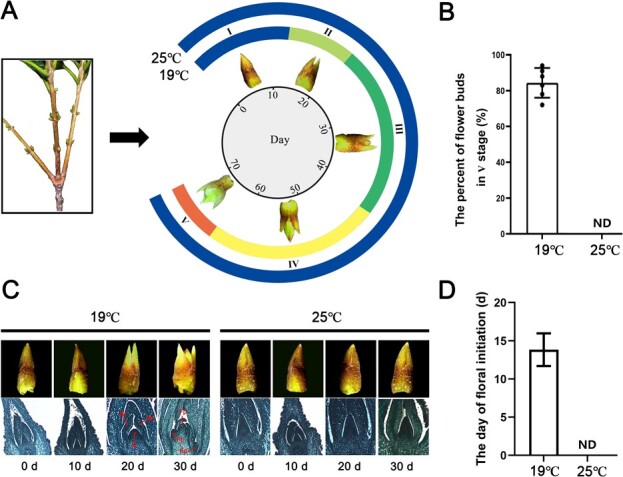
The phenotypic changes of flower bud at 19°C and 25°C in *Osmanthus fragrans*. (A) The time required for each stage of bud at 19°C and 25°C. (B) The percent of V-stage buds after 72 days at 19°C and 25°C. ND: nothing was detected. (C) Anatomical structure of buds at 19°C and 25°C during 30 days. Br: bract; Ip: inflorescence primordium; Fp: floret primordium. (D) The days required for floral initiation at 19°C and 25°C. ND: not detected.

*O. fragrans*, a woody perennial, is celebrated as one of the top 10 traditional flowers in China and has been cultivated for over 2500 years. The flowering of *O. fragrans* is significantly influenced by changes in ambient temperature, with the process being impeded under the elevated temperatures characteristic of summer [25]. In this study, we found that high ambient temperature completely inhibited the floral transition of *O. fragrans*. To gain further insight into the molecular mechanisms involved, we employed RNA sequencing to examine the expression patterns of the OfC3H gene family in response to high ambient temperature, and a differentially expressed gene (DEG) *OfC3H49* was identified. Notably, OfC3H49 was shown to function as a repressor that can directly bind to the promoter of *OfSOC1B*, thereby inhibiting flowering. Moreover, a WRKY transcription factor, OfWRKY17, has been demonstrated to bind to the promoter of *OfC3H49*, enhancing the expression of *OfC3H49* under the high ambient temperature condition. Based on these results, we propose a novel mechanistic insight into how the flowering of *O. fragrans* is inhibited by the high ambient temperatures. Specifically, a regulatory module comprising OfWRKY17 and OfC3H49 that orchestrates a delayed flowering phenotype by suppressing the expression of OfSOC1B. This study thus contributes to the burgeoning understanding of the complex regulatory networks that govern flowering in response to ambient temperatures in *O. fragrans*.

## Results

### High ambient temperature inhibits flowering of *O. fragrans*

Ambient temperature is an important regulator of plant flower induction and flowering time. To investigate the impact of ambient temperature on the flowering of *O. fragrans*, the cultivar “Sijigui” was selected for exposure to a range of ambient temperatures in climatic rooms, the flowering time was significantly delayed under elevated ambient temperatures exceeding 24°C (Fig. S1). Subsequently, we conducted a detailed examination of the phenotypic changes in flower buds at their various developmental stages under 19°C and 25°C conditions. The results indicated that the “Sijigui” flower buds was completed differentiation and flowering within 72 days at low ambient temperature (19°C), whereas this process was entirely arrested by the high ambient temperature (25°C) (Fig. 1A). An assessment of the proportion of flower buds after 62 days showed that over 84.3% of the buds had reached stage V at 19°C, in contrast to the situation at 25°C, where virtually all buds remained at stage I, indicative of foliage buds (Fig. 1B). Furthermore, histological analysis using paraffin sections revealed that floral initiation in “Sijigui” occurred within 20 days at 19°C, whereas floral initiation was significantly inhibited at 25°C (Fig. 1C, D). Collectively, these observations suggest that elevated ambient temperatures exert an inhibitory effect on the flowering process in *O. fragrans*.

### *OfC3H49* strongly responds to high ambient temperature

**Figure 2:**
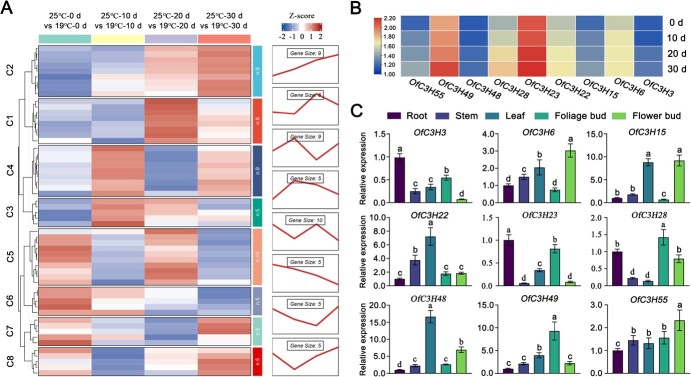
Expression profiles of *OfC3H* genes at high ambient temperature. (A) Expression trends of *OfC3H* genes during floral bud development at 19°C and 25°C. (B) Expression patterns of C2-type *OfC3H* genes at 19°C and 25°C. (C) Tissue-specific expression analysis of C2-type *OfC3H* genes by qRT-PCR.

In our previous study, we documented the involvement of the *OfC3H* gene family is involved in the flowering regulation of *O. fragrans* [26]. To deepen our understanding of the role of *OfC3Hs* role in ambient temperature-mediated flowering of *O. fragrans*, we performed RNA sequencing and analyzed the expression patterns of *OfC3H* genes at 19°C and 25°C condition (Fig. 2A, Table S1). Our analysis identified a subset of nine genes within the C2 group that exhibited responsiveness to elevated ambient temperatures, specifically *OfC3H3*, *6*, *15*, *22*, *23*, *28*, *48*, *49*, and *55* (Fig. 2B). Notably, *OfC3H23* and *OfC3H49* demonstrated a significant upregulation in response to high ambient temperatures (Fig. 2B). Tissue-specific expression analysis revealed that *OfC3H49* was abundantly expressed in the foliage bud tissue, whereas *OfC3H23* showed a higher expression level in the root tissue (Fig. 2C), indicating that *OfC3H49* may play a key role in the mediation of flowering by ambient temperature in *O. fragrans*. Subsequently, we assessed the promoter activity of the *OfC3H49* gene in response to high ambient temperature (Fig. S2). The LUC signal driven by the *OfC3H49* promoter at 25°C was significantly stronger than that at 19°C, with ~1.8-fold greater activity. Additionally, the GUS staining under the control of the *OfC3H49* promoter at 25°C appeared more intense than at 19°C in tobacco leaves and *O. fragrans* calli (Fig. S2). These results indicate that *OfC3H49* responds to high ambient temperature to regulate flowering in *O. fragrans*.

### Characteristics of OfC3H49 transcription factor

**Figure 3:**
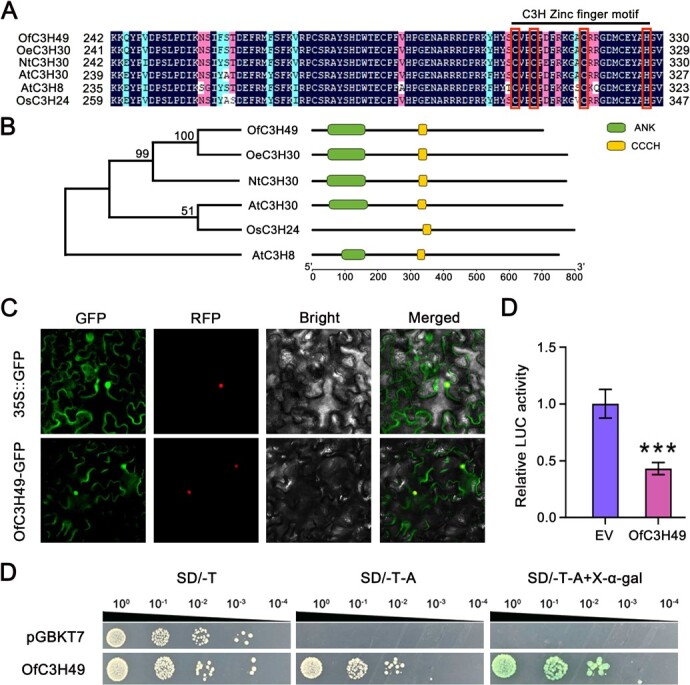
Analysis of sequence and OfC3H49 protein characteristics. (A) Multiple sequence alignment of OfC3H49 and its homologous proteins. (B) Phylogenetic analysis of OfC3H49 and its homologous proteins. (C) Subcellular location of OfC3H49. The nucleus is marked by 35S::D53-RFP. Scale bars = 30 μm. (D) Transcription repression activity analysis of OfC3H49 in tobacco leaves. The relative LUC activity was calculated by LUC/REN ratio. Data are presented as the means ± SD (*n* = 3). ^***^ indicate a significant difference compared with the empty vector (EV) at *P* < 0.001, based on Student’s *t*-test.

Sequence analysis showed that *OfC3H49* contains a 1989 bp open reading frame, encoding a protein comprising 663 amino acids (Document S1). Phylogenetic analysis showed a high degree of homology between OfC3H49 and its orthologs OeC3H30 and NtC3H30, with conserved motif of ANK and C3H domains (Fig. 3A, B). To confirm the regulatory role of OfC3H49, we further analyzed the subcellular localization and transcription activity. The subcellular localization of the OfC3H49 protein was visualized through green fluorescence protein (GFP) signals detected in the nucleus, cytoplasm, and cell membrane, suggesting that OfC3H49 protein exhibits multiple localization sites (Fig. 3C). Transcriptional activity was analyzed by measuring LUC and REN luciferase activities in tobacco (*Nicotiana benthamiana*) leaf cells (Fig. 3D). The ratio of LUC to REN indicated that OfC3H49 had significantly lower transcriptional activity compared to the control (empty vector). This result implies that OfC3H49 functions as a transcriptional repressor.

### *OfC3H49* oppositely modulates flowering of *O. fragrans*

**Figure 4:**
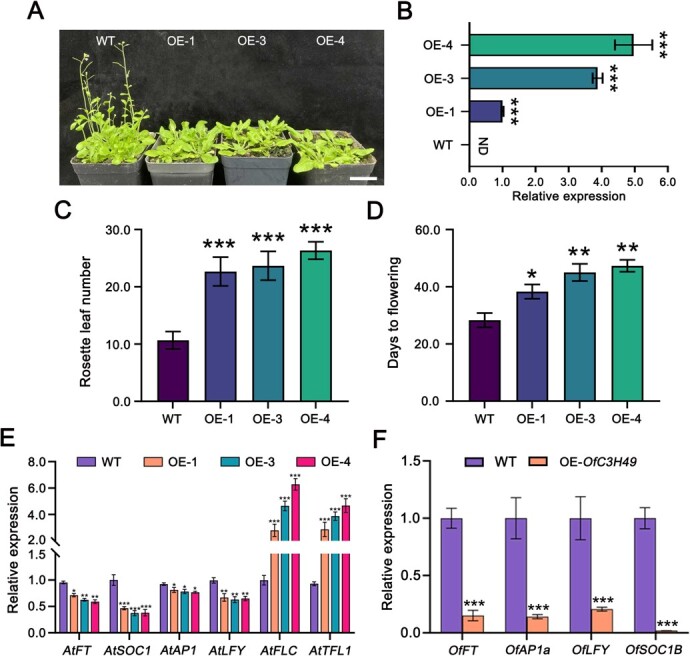
Overexpression of *OfC3H49* delays flowering in *Arabidopsis*. (A) Phenotypes of *OfC3H49*-overexpressing *Arabidopsis* plants. Scale bars = 3 cm (B) qRT-PCR identification of transgenic lines. ND: not detected. (C) Rosette leaf number. (D) Days to flowering. (E) Expression of flowering-related genes in *OfC3H49* transgenic Arabidopsis lines. (F) Expression of flowering-related genes in *OfC3H49*-overexpressing *Osmanthus fragrans* calli. Values represent the mean across three biological replicates, and error bars indicate standard deviation. ^*^, ^**^, and ^***^ indicate a significant difference compared with the WT at *P* < 0.05, *P* < 0.01, and *P* < 0.001, respectively, based on Student’s *t*-test.

To explore the role of *OfC3H49* in regulation of flowering, we introduced the *OfC3H49* gene driven by the 35S promoter into *Arabidopsis* plants and *O. fragrans* calli. Three independent *OfC3H49* transgenic *Arabidopsis* lines (OE-1, 3, and 4) were selected for phenotypic analysis of flowering (Fig. 4A, B). Our observations revealed that all transgenic lines displayed a delayed flowering phenotype relative to wild-type (WT) plants. The transgenic lines had more than twice the number of rosette leaves compared to WT plants (Fig. 4C), and the flowering time was extended by 1.5-fold in the *OfC3H49*-overexpressing lines compared to the WT plants (Fig. 4D). Furthermore, an examination of endogenous flowering-related gene transcripts revealed a significant downregulation of *AtFT*, *AtSOC1*, *AtAP1*, and *AtLFY* in the transgenic *Arabidopsis* lines when compared to their WT counterparts. Conversely, the expression of flowering repressors, namely *AtFLC* and *AtTFL1*, had high expression level in the transgenic lines (Fig. 4E). In parallel, transient transformation of the *OfC3H49* gene into *O. fragrans* calli led to a pronounced reduction in the expression of flowering-related genes, with particularly notable suppression of the *OfSOC1B* gene, as compared to the WT calli (Fig. 4F, Fig. S4). These collective results indicate that *OfC3H49* potentially exerts a negative regulatory influence on the flowering process by modulating the expression of flowering-related genes.

### OfC3H49 binds to the promoter of *OfSOC1B* and inhibits its expression

**Figure 5:**
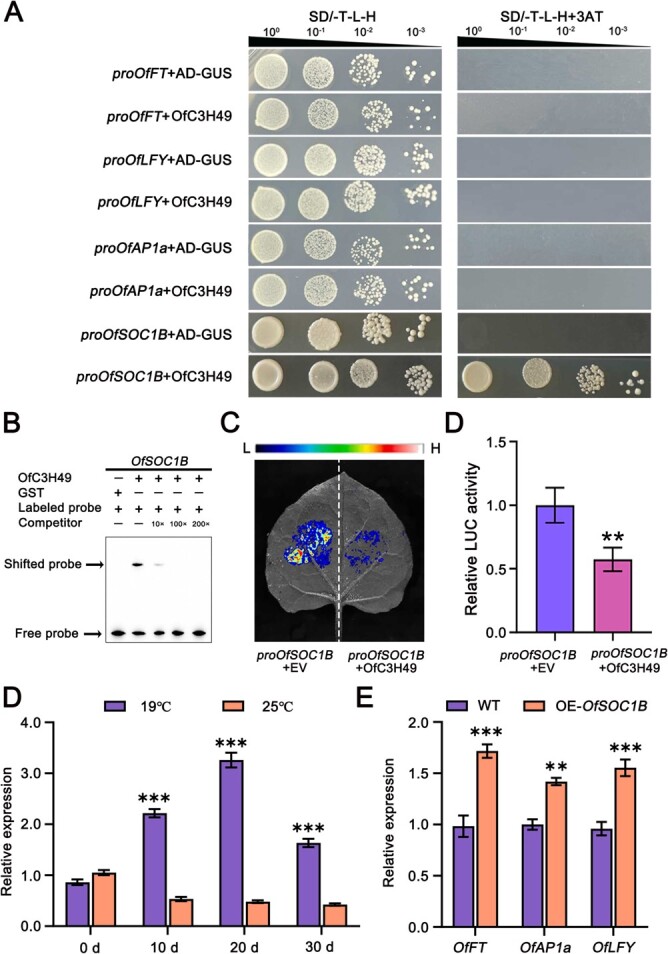
OfC3H49 directly inhibits the expression of *OfSOC1B*. (A) Yeast one-hybrid (Y1H) assay illustrating the binding ability of OfC3H49 to the promoter region of the *OfSOC1B* gene. (B) Electrophoretic mobility shift assay (EMSA) demonstrating the specific interaction between OfC3H49 protein and the *OfSOC1B* promoter *in vitro*. (C, D) Dual-LUC reporter assays in tobacco leaves confirm the repressive effect of OfC3H49 on *OfSOC1B* expression. An empty vector control (EV) containing 35S::GFP was utilized to normalize transfection efficiency. (E) Temporal expression analysis of *OfSOC1B* across various developmental stages of flower buds grown at 19°C and 25°C. (F) Expression profiling of key flowering-related genes in *OfSOC1B*-overexpressing *Osmanthus fragrans* calli. Data are represented as means ± standard deviation (SD) from three independent experiments (*n* = 3). Statistical significance is denoted by ^**^ (*P* < 0.01) and ^***^ (*P* < 0.001) using Student’s t-test.

To confirm our hypothesis regarding the direct regulatory role of OfC3H49 on flowering-related genes, we performed yeast one-hybrid (Y1H) to verify the relationship between OfC3H49 and flowering-related genes, including *OfFT*, *OfAP1a*, *OfLFY*, and *OfSOC1B*. As shown in Fig. 5A, the results demonstrate that yeast cells harboring the OfC3H49 and pro*OfSOC1B* constructs grew robustly on selective media SD/−T-L-H and SD/−T-L-H/3AT medium, confirming a positive interaction. Furthermore, EMSA analysis revealed pronounced shifts in the DNA probe fragment upon incubation with the GST-OfC3H49 fusion protein and a radiolabeled probe derived from the *OfSOC1B* promoter sequence, suggesting a direct binding affinity of OfC3H49 for the *OfSOC1B* promoter (Fig. 5B). This interaction was further characterized using Dual-LUC assay, which confirmed that OfC3H49 significantly repressed the promoter activity of *OfSOC1B* relative to the control (Fig. 5C, D). Meanwhile, we found that *OfSOC1B* expression exhibited a marked decrease in transcript levels at 25°C compared to 19°C (Fig. 5E). The functional role of *OfSOC1B* was further elucidated in *O. fragrans* by the transient transformation assays, which indicated a significant upregulation of *OfFT*, *OfAP1*, and *OfLFY* when *OfSOC1B* was introduced into *O. fragrans* calli (Fig. 5F). These results revealed that OfC3H49 delayed flowering by directly inhibiting the expression of *OfSOC1B* in *O. fragrans*.

### OfWRKY17 responding to high ambient temperature activates the expression of *OfC3H49*

**Figure 6:**
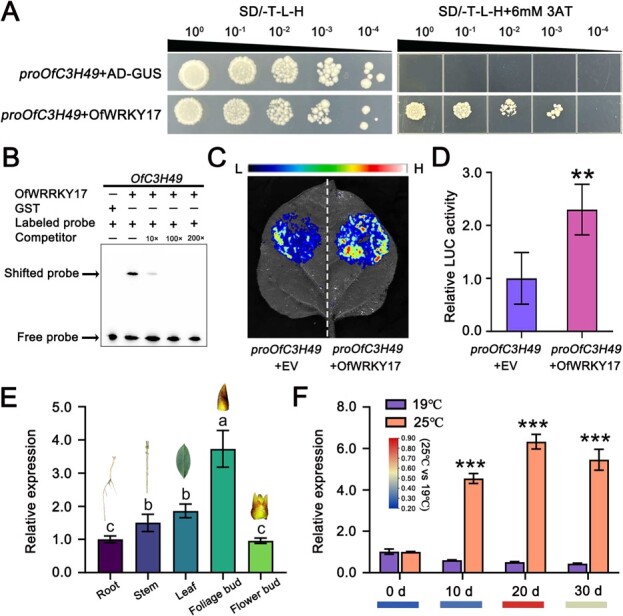
OfWRKY17 directly activates the expression of *OfC3H49*. (A) Analysis of OfWRKY17 binding to the *OfC3H49* promoter using the Yeast one-hybrid (Y1H) assay. (B) Electrophoretic mobility shift assay (EMSA) shows the interaction of OfWRKY17 with the *OfC3H49* promoter *in vitro*. (C, D) Dual-Luciferase (Dual-LUC) assays in tobacco leaves showed that OfWRKY17 activated the expression of *OfC3H49*. Empty vector (EV) 35S::GFP was used as an internal control. (E) Expression analysis of *OfWRKY17* in different tissues. (F) Expression analysis of *OfWRKY17* in flower buds at 19°C and 25°C. Different color strips represent transcriptome data. Data are presented as means ± SD of three biological replicates (Student’s *t*-test, ^***^  *P* < 0.001).

To elucidate the molecular mechanisms underlying the regulation of OfC3H49, we conducted Y1H screening to identify potential upstream regulators. In our screening, the WRKY transcription factor OfWRKY17 was identified, which served as a direct interactor with the promoter of *OfC3H49* (Fig. 6A). Previous studies have reported that WRKY transcription factors bind to W-box elements in the promoters of their target genes [ 27], we conducted EMSA probe to detect the combining ability. The EMSA results confirmed that OfWRKY17 could bind to the *OfC3H49* promoter by the TTGAC site (Fig. 6B). Dual-LUC analysis substantiated this regulation, demonstrating that OfWRKY17 activates the *OfC3H49* promoter (Fig. 6C, D). Subsequent analyses focused on the role of *OfWRKY17* in the flowering regulation of *O. fragrans*. Expression profiling indicated that *OfWRKY17* is highly expressed in foliage bud tissue, the expression of *OfWRKY17* is significantly induced by 25°C relatives to 19°C during the floral transition of *O. fragrans* (Fig. 6E, F). In addition, the OfWRKY17 characteristics of transcription activity and subcellular localization were analyzed (Fig. S3). The transcriptional activity of OfWRKY17 was assessed in yeast cells, where growth on selective medium containing X-α-gal confirmed its activation capacity (Fig. S3A). The OfWRKY17-GFP fusion protein was transiently expressed in tobacco leaves, with confocal microscopy revealing nuclear localization (Fig. S3B). Furthermore, the promoter activity of *OfWRKY17* was evaluated under 25°C and 19°C condition, paralleling the behavior of *OfC3H49*, with both LUC and GUS activities being significantly induced by high ambient temperature (Fig. S3C–F). Collectively, these results indicate that the OfWRKY17 transcription factor can respond to high ambient temperatures to regulate the expression of *OfC3H49*.

**Figure 7:**
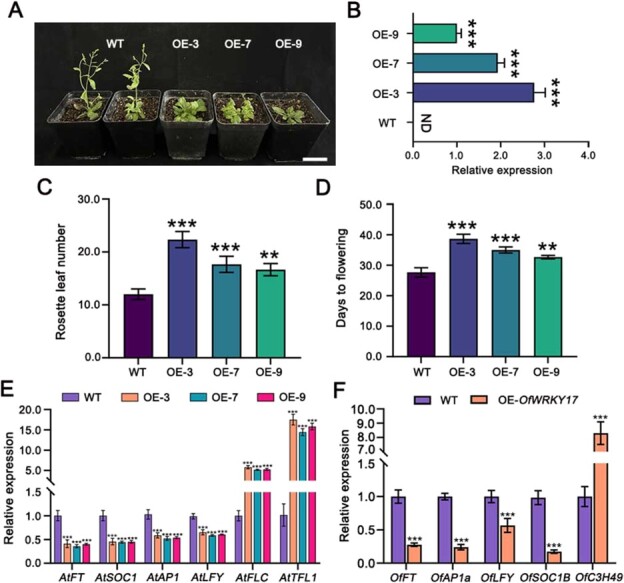
Overexpression of *OfWRKY17* delays flowering in *Arabidopsis.* (A) Phenotypes of *OfWRKY17*-overexpressing *Arabidopsis* plants. Scale bars = 3 cm (B) qRT-PCR identification of transgenic lines. ND: not detected. (C) Rosette leaf number. (D) Days to flowering. (E) Expression of flowering-related genes in the *OfWRKY17*-overexpressing *Arabidopsis* lines. (F) Expression of flowering-related genes in *OfWRKY17*-overexpressing *Osmanthus fragrans* calli. Values represent the mean across three biological replicates, and error bars indicate standard deviation. ^*^, ^**^, and ^***^ indicate a significant difference compared with the WT at *P* < 0.05, *P* < 0.01, and *P* < 0.001, respectively, based on Student’s *t*-test.

To ascertain the role of OfWRKY17 in the regulation of flowering, *OfWRKY17* was overexpressed in both *Arabidopsis* and *O. fragrans* calli (Fig. 7). The overexpression of *OfWRKY17* significantly delayed the flowering time in transgenic *Arabidopsis* plants (Fig. 7A, B). Transgenic lines exhibited more than double the number of rosette leaves compared to wild-type (WT) plants, and the interval to flowering was ~1.3 times longer than that of WT plants (Fig. 7C, D). In these transgenic plants, the expression of flowering genes (including *AtFT*, *AtSOC1*, and *AtAP1*) was significantly downregulated, whereas the gene of *AtFLC* and *AtTFL1* was markedly upregulated relative to their WT counterparts (Fig. 7E). Furthermore, the overexpression of *OfWRKY17* in *O. fragrans* calli led to a significant upregulation of *OfC3H49* expression and a concurrent inhibition of flowering-related genes (Fig. 7F).

## Discussion

### *OfC3H49* delays flowering of *O. fragrans* by inhibiting the expression of *OfSOC1B*

Against the backdrop of global warming, ambient temperature is steadily increased and the elevated temperature significantly affect the developmental and physical process of plants [28]. In this study, we found that high ambient temperature (more than 24°C) acted as a flowering repressor that completely inhibited the floral transition of *O. fragrans* (Fig. 1). This is in concordance with observations in Olive (*Olea europaea*), where high temperatures suppress the expression of *OeFT1/2*, resulting in non-flowering [29]. In contrast, the increase of ambient temperature has a positive effect on *Arabidopsis* flowering [30]. These disparate responses underscore the diversity of adaptive mechanisms that plants have evolved in relation to ambient temperature variations across different species. The C3H family of transcription factors has been implicated in the response to changes in ambient temperature and in the regulation of temperature-mediated flowering processes in plants [31, 32]. Through the transcriptomic analysis, we identified a member of the *OfC3H* family gene, *OfC3H49*, which showed the most pronounced response to high ambient temperature (Fig. 2). We further investigated the role of *OfC3H49* in the inhibition of flowering in *O. fragrans* under high ambient temperature.

Phylogenetic analysis showed that OfC3H49 is homologous to *Arabidopsis* AtC3H30/ (Fig. 3A, B). AtC3H30 has been proved to directly activate the expression of *AtSOC1* and *AtFT* to promote flowering under conditions of high stress intensity [33]. In the absence of stress, AtC3H30 contributes to the maintenance of vegetative growth by suppressing the expression of flowering-promoting factors [34]. Consequently, we hypothesize that the OfC3H49 protein may play a pivotal role in the regulation flowering. Then we analyzed the characteristics of OfC3H49, OfC3H49 acts as a transcriptional repressor, which exhibits a multi-localization pattern, being detected in the nucleus, cytoplasm, and cell membrane (Fig. 3C), similar to other C3H proteins such as OsTZF1 [35] and AtTZF1 [36]. Meanwhile, overexpressing *OfC3H49* in *Arabidopsis* plants displayed delayed flowering phenotypes, characterized by an increased number of rosette leaves and extended days to flowering relative to WT plants (Fig. 4A–D). The expression investigation revealed that the flowering-related genes (*FT*, *SOC1*, *AP1*, and *LFY*) were significantly downregulated in the *OfC3H49*-overexpressing *Arabidopsis* lines and *O. fragrans* calli (Fig. 4E, F), which explained the phenotypes of late-flowering in *Arabidopsis* plants. Comparable observations have been reported for other *C3H* genes, overexpression of *AtZFP1* and *MsZFN* in *Arabidopsis* has been shown to significantly reduce the expression of *FT* and *SOC1* [37, 38]. Guided by these clues, we utilized Y1H assay to screen the interaction between OfC3H49 and flowering-related genes. The evidences indicate that OfC3H49 can directly bind to the *OfSOC1B* promoter and inhibit its expression (Fig. 5), which is similar to the homologous protein in *Arabidopsis* [34]. These findings suggest that OfC3H49 delays flowering in *O. fragrans* by directly inhibiting the expression of *OfSOC1B* under high ambient temperature.

### OfWRKY17-OfC3H49 module responding to high ambient temperature negatively mediates flowering of *O. fragrans*

WRKY transcription factors constitute a substantial gene family that orchestrates plant growth, development, and responses to abiotic and biotic stresses [39]. Emerging research underscores the involvement of WRKY proteins in determination of flowering time through their interaction with W-box elements in the promoters of their target genes. For example, *Arabidopsis* AtWRKY71 facilitates flowering through directly binding to the *FT* promoter and enhancing its transcription [ 40], AtWRKY12 and AtWRKY13 delay flowering by directly downregulating the expression of *FUL* under short day conditions [37]. Yang *et al.* [38] found that BnWRKY184 as a positive regulator of flowering, which upregulates *AtFUL* expression in transgenic *Arabidopsis* plants. In *O. fragrans*, we found that OfWRKY17 could directly bind to the promoter of *OfC3H49* and activate its expression (Fig. 6A–D). Certain research has shown that *WRKY* genes response to temperature stress such as heat and cold stress [41, 42], while there few reports on WRKY how to response to changes of ambient temperature. In our investigation, we noted that the expression and promoter activity of *OfWRKY17* were markedly induced by 25°C relative to2 19°C (Fig. 6E, F; Fig. S2, and Fig. S5), indicating that OfWRKY17 involved in the responding of high ambient temperature in *O. fragrans*. To confirm the role in flowering regulation, *OfWRKY17* was overexpressed in both *Arabidopsis* plants and *O. fragrans* calli (Fig. 7). The *Arabidopsis* lines overexpressing *OfWRKY17* exhibited delayed flowering compared with WT plants (Fig. 7A–D). Meanwhile, the expression of flowering-related genes was decreased in *OfWRKY17*-overexpressing *Arabidopsis* and *O. fragrans* calli (Fig. 7E, F). All the results revealed that *OfWRKY17* responding to high ambient temperature negatively regulates the flowering of *O. fragrans* by activating the expression of *OfC3H49*.

**Figure 8:**
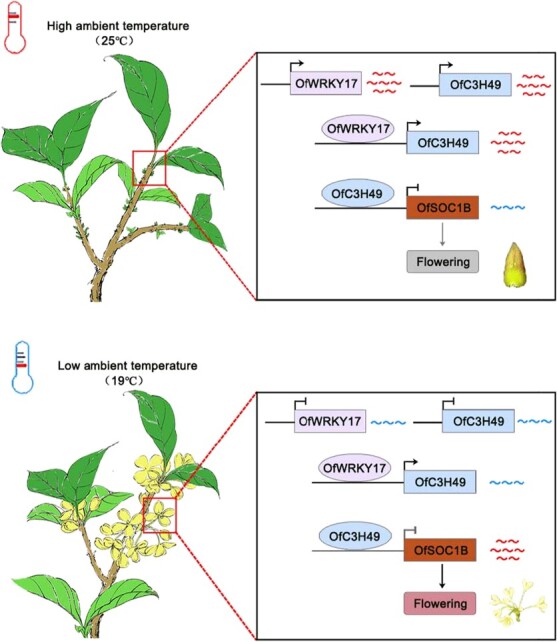
Proposed working model of OfWRKY17-OfC3H49 responding to ambient temperature regulates flowering by inhibiting *OfSOC1B* expression in *Osmanthus fragrans*.

Therefore, we propose a model that illustrates the role of the OfWRKY17-OfC3H49 module in ambient temperature-mediated flowering and its regulation of *OfSOC1B* expression in *O. fragrans* (Fig. 8). Under condition of high ambient temperature, the expression of *OfWRKY17* and *OfC3H49* are induced, inhibiting the expression of *OfSOC1B*, thereby suppressing the flowering of *O. fragrans*. In contrast, the expression of *OfWRKY17* and *OfC3H49* is reduced under condition of low ambient temperature, leading to the upregulation of *OfSOC1B*, which in turn promotes the flowering of *O. fragrans*.

## Conclusion

In this study, we have identified a C3H gene, *OfC3H49*, in *O. fragrans* that is induced by high ambient temperature. *OfC3H49* negatively regulates the flowering process of *O. fragrans* by suppressing the expression of *OfSOC1B*. Furthermore, the transcription factor OfWRKY17 was found to respond to high ambient temperature and promote the expression of *OfC3H49* in *O. fragrans*. These insights not only contribute to the understanding of the molecular basis of ambient temperature-responsive flowering but also highlight the potential for *OfC3H49* as a key gene in flower regulation of *O. fragrans*.

## Materials and methods

### Plant materials and treatments

The eight-year-old potted *O. fragrans* “Sijigui” plants were preserved in a greenhouse of Zhejiang Agriculture and Forestry University (Hangzhou, China). To explore the effect of high ambient temperature on the floral transition of *O. fragrans*, six uniform “Sijigui” plants were placed in an artificial climatic room at 25°C, with a 12/12-h photoperiod. The control group was grown at 19°C in an artificial climatic room, with the same photoperiod. After 0, 10, 20, and 30 days of treatment, the buds of same stage were collected at 19°C and 25°C, respectively. Additionally, the tissues including root, stem, leaf, foliage bud, and flower bud were collected under natural conditions.

### Phenotype investigation

To investigate the effect of high ambient temperature on the floral transition of *O. fragrans*, the phenotypic changes of buds under ambient temperature treatments were observed. According to Wang *et al.* [25] method and phenotype changes, the development stages of floral bud were divided into five stages (named I to V stages). Total 100 undifferentiated foliage buds were labeled on “Sijigui” plants at 19°C and 25°C, and the phenotypes of buds at different stages were observed using a stereomicroscope (Stemi 508, Zeiss, Germany). The time required for each stage of buds and the number of buds that eventually reached stage V were counted at 19°C and 25°C, respectively.

Additionally, the anatomical structure of buds was observed by paraffin section. At least 10 similarly sized buds were fixed with formaldehyde-acetic acid-ethanol fixative (FAA) solution, and the buds were then dehydrated with gradient ethanol and gradient xylene. Subsequently, the samples were immersed in paraffin at 60°C for three days, and then embedded in paraffin blocks. A slicing machine (LEICA RM2235, Leica, Germany) was used to obtain ~8 μm slices of materials, which were dewaxed in xylene and stained with hematoxylin and fast green FCF, and were observed using a vertical fluorescence microscope (Axio Imager A2, Zeiss, Germany).

### RNA sequencing

Total RNA of samples was obtained using a FastPure Plant Total RNA Isolation Kit (Polysaccharides & Polyphenolics-rich) (Vazyme, Nanjing, China), and its quality was counted by NanoDrop One (Thermoscientific, Massachusetts, American). Illumina Novaseq 6000 platform was used for RNA sequencing, and gene expression was calculated by the fragments per kilobase million (FPKM) method. DEGs were selected by the standard of |log_2_FC| ≥1 and false discovery rete < 0.05. Heatmaps of gene expression were constructed using the TBtools software (v2.083) [43].

### qRT-PCR analysis

First-strand cDNA was synthesized using HiScript III All-in-one RT SuperMix Perfect for qPCR (Vazyme). qRT-PCR analysis was performed on Lightcycle 480 system using a Taq Pro Universal SYBR qPCR Master Mix (Vazyme). *OfActin* (*O. fragrans*) [44] and *AtActin* (*Arabidopsis*) [45] were used as internal control. Both three biological and technical replicates were performed on each sample. The primer sequences of qRT-PCR were provided in Table S2.

### Sequence analysis

The coding sequence (CDS) of *OfC3H49* (Genebank: PP108735) was amplified from the foliage bud tissue of *O. frgarans* “Sijigui” using a 2 × Phanta Flash Master Mix (Vazyme) according to manufacturer’s instructions. Multiple sequence alignments of OfC3H49 and its homologous proteins was performed using DNAMAN 7.0. Phylogenetic tree analysis was analyzed by MEGA 11.0 using the neighbor-joining method with 1000 bootstrap iterations.

### Subcellular localization

The CDS of *OfC3H49* and *OfWRKY17* without the stop codon were integrated into *Bam*H I and *Xho* I sites of the vector pORE-R4-35SAA [46] driven by the 35S promoter to produce OfC3H49-GFP and OfWRKY17-GFP fusion plasmids. Recombinant vectors OfC3H49-GFP, OfWRKY17-GFP, 35S::GFP (as the control), and the 35S::D53-RFP (nucleus marker) were transferred into *Agrobacterium tumefaciens* (*A. tumefaciens*) GV3101 strain and then co-injected into tobacco leaves. After dark incubation for 48 h, the fluorescence signals were observed using a laser scanning confocal microscope (Olympus Corporation, Tokyo, Japan).

### Transcriptional activity analysis

To explore the transcriptional repression activity of OfC3H49, the CDS of *OfC3H49* was cloned into the *Bam*H I and *Xho* I sites of the pGreenII-62sk-GAL4 vector [47]. Recombinant plasmid GAL4-OfC3H49, empty vector (as the control), and pGreenII 0800-LUC-TATA were transformed into *A. tumefaciens* GV3101 (pSoup) strain, and then co-injected into tobacco leaves. After dark incubation for 48 h, the LUC activity was measured using a dual-luciferase reporter assay system (Promega, Beijing, China). To investigate the transcription activation activity of OfWRKY17, the CDS of *OfWRKY17* was inserted into pGBKT7-BD vector with *Eco*R I and *Bam*H I sites to produce pGBKT7-OfWRKY17 plasmid and was introduced into the yeast AH109 strain using the PEG/LiAC method. The transformants were grew on the synthetic defined (SD) medium without tryptophan (SD/−T), and then transferred to SD medium lacking tryptophan and adenine (SD/−T/−A), and SD/−T/−A + X-α-gal medium to observe yeast growth at 30°C for 3 days.

### Promoter activity analysis

The *cis*-acting regulatory elements (*CREs*) in the 2000-bp promoters of *OfC3H49* and *OfWRKY17* were identified and analyzed using PlantCARE (https://bioinformatics.psb.ugent.be/webtools/plantcare/html/). The promoters of *OfC3H49* and *OfWRKY17* were inserted into the *Bam*H I and *Xho* I sites of the pGreenII0800-LUC vector and the *Hin*d III and *Bam*H I sites of pCAMBIA1300-GUS vector, respectively. As previous described, the recombinant plasmids *proOfC3H49*-LUC, *proOfWRKY17*-LUC, *pro OfC3H49*-GUS, and *proOfWRKY17*-GUS were transformed into tobacco leaves and *O. fragrans* calli via *A. tumefaciens*-mediated transformation [48]. After 3 h treatments of 19°C and 25°C, the LUC signals of transgenic plants were observed with a FUSION FX EDGE SPECTRA (VILBER BIO IMAGING, Paris, France) and quantified by a dual-luciferase reporter assay system (Promega). Additionally, the GUS activity of transgenic plants was determined using a GUS staining Kit (Coolaber, Beijing, China) after 12 h of treatment at 19°C and 25°C.

### Generation of transgenic *Arabidopsis* and *O. fragrans* calli

Transgenic Arabidopsis plants of *OfC3H49* and *OfWRKY17* were generated by the floral-dip method [49]. The flowering phenotypes of T_3_ generation transgenic *Arabidopsis* plants were observed, including the time required for flowering and the number of rosette leaves. Additionally, the expression of endogenous flowering-related genes (*AtFT, AtFLC*, *AtTFL*1, *AtSOC1*, *AtLFY*, and *AtAP1*) was measured by qRT-PCR when Arabidopsis was just flowering. Ten plants from each line were used in this study.

For transient transformation of *O. fragrans* calli, the fusion constructs 35S:*:OfC3H49*, 35S::*OfSOC1B*, and 35S::*OfWRKY17* were separately transfected into *O. fragrans* calli using the method of Zhong *et al*. [52]. After incubation for 3 days under dark conditions, the expression of flowering-related genes (*OfFT*, *OfAP1a*, *OfSOC1B*, and *OfLFY*) were determined by qRT-PCR. Ten separate *O. fragrans* calli were used for each gene in this study.

### Y1H assays

A Y187 system was used for Y1H assays. The CDS of *OfC3H49* was inserted into the *Eco*R I and *Bam*H I sites of the pGADT7-AD vector to produce pGADT7-OfC3H49 constructs. In addition, the promoters of *OfFT*, *OfLFY*, *OfAP1a*, and *OfSCO1B* were cloned into the pHis2 with *Eco*R I and *Sac* II sites. The pGADT7-OfC3H49 and empty vector pGADT7-AD were separately transformed into yeast strain Y187 using the PEG/LiAC method with pHis2-*proOfFT*, pHis2-*proOfLFY*, pHis2-*proOfAP1a*, and pHis2-*proOfSCO1B*. Yeast cells were plated on SD plates without tryptophan, leucine, and histidine (SD/−T-L-H). Positive clones were transferred into an SD/−T-L-H + 3AT medium for stringent screening of possible interactions.

The cDNA library of *O. fragrans* “Sijigui” was constructed by BioGene Biotech (Shanghai, China). The promoter of *OfC3H49* was inserted into the bait vector pHis2 and then transformed into the Y187 yeast strain. The colonies were selected on SD/−T/−L/-H + 3AT medium. After determining the minimal inhibitory concentrations of 3AT for the bait strains, the linear pGADT7-Rec vector was cotransformed into the bait yeast strains and selected on SD/−T/−L/-H + 3AT agar medium. The genes obtained from Y1H screening were further verified by point-to-point experiment to confirm DNA-protein interactions.

### Dual-LUC assays

The promoters of *OfSOC1B* and *OfC3H49* were inserted into the pGreenII0800-LUC vector to produce *proOfSOC1B*-LUC and *proOfC3H49*-LUC reporters. The 35S::OfWRKY17 and 35S::OfC3H49 plasmids were used as the effecters. Following the method of Wang *et al.* [25], Dual-LUC assays were performed. The LUC signals were observed by FUSION FX EDGE SPECTRA (VILBER BIO IMAGING). Dual-LUC assays were independently repeated three times.

### EMSA assays

The *OfWRKY17* and *OfC3H49* CDS sequences were inserted into the pGEX-6P-1 vector with *Eco*R I and *Xho* I sites. Fusion proteins GST-OfWRKY17 and GST-OfC3H49 were purified from *Escherichia coli* (*E. coli*) strain C43 (DE3) cells using GST Tagged Protein Purification Kit (Biolinkedin, Shanghai, China). The DNA probes containing “AATTGACTTGA” (WRKY binding site) and “CAAAAAGTAAT” (C3H binding site) were labeled with biotin. Additionally, unlabeled DNA probes were used as competitors. EMSA assays were conducted by Chemiluminescence EMSA Kit (Beyotime, Shanghai, China). The probe sequences used in this study were listed in Table S2.

### Statistical analyses

All experiments were independently repeated with at least three times, and were represented as the mean ± SD. Statistical analysis was performed by SPSS 17.0 with one-way ANOVA and Student’s *t*-test. Significance was indicated by ^*^  *P* < 0.05, ^**^  *P* < 0.01, and ^***^  *P* < 0.001.

## Supplementary Material

Web_Material_uhae273

## Data Availability

The RNA sequencing data have been submitted to the NCBI Sequence Read Archive (SRA) under the BioProject accession number PRJNA961323. The CDS of *OfWRKY17*, *OfC3H49*, and *OfSOC1B* has been deposited in the National Center for Biotechnology Information (NCBI) with the accession numbers PP108736, PP108735, and PP103737. Other data supporting our findings are available in the manuscript file or from the corresponding author upon request.
