# Supplementary material for: OfWRKY17-OfC3H49 module responding to high ambient temperature delays flowering via inhibiting OfSOC1B expression in Osmanthus fragrans
Source: Hortic Res. 2024 Sep 24;12(1):uhae273. doi: 10.1093/hr/uhae273 (PMC11725642; doi:10.1093/hr/uhae273)
Supplement: Web_Material_uhae273 [file web_material_uhae273.zip › Document.docx]

**Document S1.** The CDS of *OfC3H49* gene.

ATGTGCAGTGGCCCAGAGCAATCAAATACAAGTTCAACTGCCACGCAATCCGCCATGGCTACCAAATCAAATAACATGGACACAAGTAATCTAACCGTAGAAACTGAAGATTCCTTTTCTAGTCTACTTGAATTTGCTGCCAACAATGATGTCGAGGCATTCAAGAGATCCATTGAGCTTAATTTGTCTGCAGTTGACGATGTTGGTACTTGGTACATTCGCAAGAAGGGTTCAAAGCAGATCACACCGGAGGAAAGAACCCCCTTGATGGTTGCTGCTACCTATGGTAGTGTTGATGTTTTGAAACTGATGGTTGCTCTGCCTGAGGTTGATTTGAATAGAATATGTGGTAGAGACAAGTGTACTGCCCTCCATTGTGCTGCCTCTGGTGGCTCCGCTCTTTCCTTTGAAGCCATCAAGCTGCTACTCTCGGCTGGTGCAGATCCAAATATTGAAGATGCAAACGGTCATCGCCCAGTTGATGTGATTGTTGTTCCTCCTAAACTTCTGGGTGTTAAAGCTGCTCTTGAAGAACTGCTTGTGAACAATATTTCTGATGGATCACTTGGCGAGTACAATTTAAGGGTTTCAATATCTAACTCCACTGCTTCTTCACCTGTTCTATCTTCTTCCCCAGAAAGTAGGTCTCCAGGTTCACCATCGGATTCAGCATCATCTCCAATGGCATCTAAGTCGAATGATGTCCCTGCTAAATCTATGTCAGAGAAAAAGCAATACCCGGTGGATCCATCTCTCCCTGACATCAAGAATAGCATTTTCTCAACTGATGAGTTCCGGATGTTTTCATTCAAGGTCAGGCCTTGTTCAAGGGCCTACTCTCATGATTGGACCGAGTGTCCATTTGTTCACCCTGGAGAGAATGCTCGCAGAAGAGACCCACGAAAGTACCATTACAGCTGTGTCCCTTGTCCTGATTTTCGTAAGGGTGCCTGTAGGCGAGGGGATATGTGTGAGTATGCTCATGGTGTGTTTGAGTGCTGGCTACACCCAGCTCAGTATCGGACAAGACTGTGTAAGGATGGCACAAGCTGTGCTAGACAAGTGTGTTTCTTTGCACACACCCCAGAGGAGCTTCGACCCCTTTATGTTTCTACTGGATCTGGTGTTCCTTCACCCAGGTCAGCTGCCTCTGCAGCTAGTGTCATGGACATGGCTGCAGCTTTGAGCCTTTTGCCTGGGTCACCTTCTTCACACTCTGTCATGTCTCCTGCATACAAACAACCCATGTCTCCCACTGCAAATGGGATGTCTCATTCATCTGCAGCTTGGCCTCAACAAAATGTCCCGACCCTTCATCTCCCTGCTAGCAACATCCAATCCAGCCGTCTCAGATCATCTCTTAGTGCCCGAGACATTCTACCTGAGGATTTGAGCATGTTGCAGGATTTTAATGCTCAGCAACATGTTTTGAATGACTTGGCTTGTTTTTCTCAGCCACGTCCTAATTCTGGTACTTTGAATCATTCTGGTCGGTCCAATACACTAACACCTTCCAATCTGGAAGAGCTATTTTCTGCGGAGATCACCTCGTCACCAAGATATTCTGATCAGGCAGTGGCTTCTGGTGCATTTTCTCCTAAGCACAAATCTGCTGTTCTCAATCAGTTTCAGCAGCAACAGAACATGCTTTCACCAGTCAATACTAACATATTTTCCCCTAGAAATGTTGAACACTCTTTGTTGCAGGCTTCCTTTGGGGTCTCATCACCAAGGATGTCGTCACCAAGAAGTGTGGAACCCTCGTCACAAATGAGTGCCAATGTTTCAACCTTTGCTCAACGAGAGAAGCAGCAGCAACAGCTGCGTGTCCTCAGTGATATTGCTCATATGAAAAGATCAACTTCCGTTGAACCCAATAACGAGGGAGAGGAGCCTGATCTGTCGTGGGTACAATCTCTTGTCAAGGAATCACCACCTGAGATGGAGAAGCTTTTCGGCTCCCATTTCTGGTGCTGCCCCATCCGGTGA

**Document S2.** The promoter of *OfC3H49* gene.

CAACAACATACTAACATCGTTATTTATGCGAATGGCCTTTTAATAAGCATATATCTAATTGACTTGAAATAAATGAATTATTGAATCACTATTAAAAATAAAATCAGAAAAAAAAAAAATTCTTATTGGTACAATAATTTGTCGAACTAATACTTAGGGCTTGTTTGTTTTAGATTAAAATATGATTTTAATCTAAATCAAAGTCAAATAATCAATATCAATCGCATATTTCTCGAGTCCCACAACTTTTTAACTATGCATTTCAAAAGCTTCTATCACAATCTACTAAAATTCTAATTTTAATTCTTATCAAATACACTCTTAACGCATAAGAGCAAAAAATTCACCGCAACTTATTTTTCATTGACTGGTCACTCTTTACAAGAGGCGAGTTAATGATGAAATTCAACTTCCTTGTGTTTAATTGGATTCGAAAGATTTTTTTTAGTAAATTGCTTTATTCATTCCTTTGTCCAATTGGACTCCATTAAAATGAGATATGGGGAGAAAAAGTGTTTCAAAATATTTATTTTTTTAGAGAAGATGTAGGTAAAAAAAAAAAAGTTTTGATTTTACGTATAATATTAGTTTATGAAAAATTATTTGGTTGAAAATGAGTTTGGAAAAAATGGAGCCCAATAGGGGCTAAATTTAAAGAAAAGAAAAGTGACTCGAGTGAAGAAAAAGTAGAAATAATAAAAAGAAAAAGGGCATCCAAACGCGGCCTAGCTCCTACCGGCCAAAACCCCACTATCAAATATCTTCTTAATTTGACTGGCATTTACATTTCATTTCGCGTAAATTTCAACATATTTGCATTTGCATTCGCATTTCATTAAATTTCATTTCAATTTCAATCCGTTCCTCCACCGCTCTAGTACACACACTTGCACCCTGCCTTCCTGCAGCAGAGAACAAGGCCCATATATCGCTTTCTCTCAATCTTTCTTCTCCCTCCTATACATACATACGCACGCTCCCCCTAAATCTCCGTATGGGTACCTGAAACTTCGTTTCTTATCTATTTTTTTTATTAATTGTTATCTGTATGATAAAGATTTCTTCAGTGGGAACTCCCTTTGTTACAAGCTTTGTTTCTTATTTTCTTCTTCAAGTTCAAACCGAAGATCGTTAATTAGTGACTGACCCATTTACAGATCTTGTAAAAAATCTTCTTTTCGGATCATCGGTAAGTTTTATTTAGTGGGCAGGTTTTCTTACTTCGCTTGGGTATACTTTGATTTTCAGTCGGAATATGTGAACTAAAGAAATGATTATTTTAGTATAGAGATTCGATTTAATTTTGTAGATCTGTGCTTCTTTGTATGATTTTGAATGAATATAAATATAGGATACACTCGAATGTTTCTTAGTGTTTACGTGCATATATATCTTTGCTTTTAAAATTTTGAACTTTTTGAGATATGTGTAATTAATTATAGGTACTAGAAATATGAATATTTTTCTATATCTTGTTTGTTTTTGCGAAAGATTTTTGTATTTGACAATGATTTTCTTCCCCTAAATTTGTTCTAATAAGTTTTTGTCTAGCTGATAGAAAAATTGGCCGGAGCAGTATCTAATTCCTTTGGGGAAGTAGGGATATGATTTTTTTTTTTTAACCATGTATGAGAGAAAAAATCTCTGAAGCCTTGTGTAATTGGATTGATTTTACTTGTTGAACAAGTTGTTTGGATTTTGTTTTGATCTTGAGGGTTTGAAGTTTATGACTAAATTTAATGGTCTGGCTGCAATAATTGTGCCGGAATTGGTGAAGAAGAGCCTGTGAATTGTTATTATTCAATATTTACCTTTTACTCTCAAGATTATGATTTTATAATATAAATGAATTTTAACATTTTCAGAGCTTAATGAGAATGAATGAATCATGAAGGAGTTTCTTTTTAAGAATACTGTTTTAATTTCTTGTTTTCGAGTTGGGTTTATAATGATCTTGATCTAAAAAACGCAGTTCTTGATTGACTGCAGCTGATTTAAGT

**Document S3.** The CDS of *OfSOC1B* gene.

ATGGCGAGGGGAAAGACTCAGATGAGGCGTATAGAAAATGCAACAAGTAGGCAAGTGACCTTCTTCAAGAGGAGAAATGGGCTTCTCAAAAAGCTTTTGAACTTTCAGTCCTCTGTGATGCTGAGCATGAAGGAGACAATAGAACGTTATAAAAGGCATAAAAAAGATATTCAGGCTAAGAACCCTCCAGTGGAACAGAACACGCAGCATTTACAGCACGAAATGGCAAGTCTGATGGAAAACATAGAGCGTCTCGAATCTTCAAAAAGGAAACTCTGGGGGGAAGATCTGGGATCATGCACCAATGAAGAACTACAAGAGCTAGAACAACAGCTGGAGCGTAGTTTCAGCAGCATTCGAACAAGAAAGATTCAAGTTTTCAGGCAACATATAGAGCAATTGAAAGAAAAGGGAACAGCTTTAGCAGCTGAAAATGCAATGTTGTGGGATAAGCTTTCACTCCAACAAGAACGAGGATCAAAGGAGGAAAGAGCAATTGAGCCCTCTGCAGATATTAGTGAAGTTTCAGTTGTGGAAACAGGCTTGTTCATTGGACCTCCAGAAACCAGTGAGAATCGTCCCCTACAGAAGCCATAG

**Document S4.** The promoter of *OfSOC1B* gene.

ATTTGTCATTTGTATCCTTCTCTACTTCGATCATTTTACTTTGTAATATACACCCGGAAGGTTTGCTAGACAACATTTGCCTAAGTTTGTACTTAGTGTTCGAATTCACGAATCTTCACATCGCAATTGGAATGTTGCTGCAAACTTAAGAGCAAATGGTGAAATAGATGTCTCGCTATAGGTTGGGCCTATTATATTAGAGAGAAGCAACTATAAATGTGTGACACATGTCTTTGAGCAACTAAATAACGAACCCCAAATGTTTATGGTTTACAAAACAAGCCACACTGATGGAACTCTGACTTGCCCTTGAACTATTAAATTTTAGTTGTATCTTTTCCATTTAGCTAATATGAAACAATTCTCTTTCCCTATGTACTAAAAATTCATCACTGGATCTTTGTTCTTAATTTAGTCATGCTATTTCTAATTCTAATTCCATCCGCTTTCGTGTTTACTTATATTGAAGTATATCGCTTAATTGATTATATTATCAATTCATTCTTTCATATACATACAAGTAAGTTTCGATTGTTTAAAATTAAACAATCGCTTTAACTCTTTCATACCTCATAGAATGGATGTACCATACCGTTTCTTTCGAATTATCACACCTTCTTTGGGACATGACATTCATCTGATGAGTTCATCTTTATAAACACCAAAAAAACCTTTGAACACAAATTTGCGACCCATTTTTTTCTTCTCAAACACTTACATCTCTTCTATGTCAACTTGCACGTGCGTGCTTTATTCAATTTTAGTTTATGGAGTACTGTTGTACATGTCCGTTGAACAATCACCATACGCTTATATTTAACATTCATCGACTAACGTACAATTCACATTTGCACGTTTTCTCACTAGTATGCAAGAAAAGGAAAGAAAGAGCAAGAAGCTTCTTATATGAATAAGTAAGAAAACAAGTGTGTGTGAAACAGAAAATCTTCTAGGGGGTTGTGCAAAGGACATCTAGTAATTGAAGCCACAGCTCTAGCTCCAACTTGAACACCGGAAAAAGAGACGAAAATATCACCACCCCATTAAAGACAGAGAATAGGTACAAAAAGGCCATTTCTATCTTAAGGCCCTTCTCATGGACCGTATGTTGTATTTTTTGCTCATACTATGAACTTTCAATTATTTGGTTTTTGGGCTTTTACTCTATCCAATGACTCTTCTCAAGAGAAACAGAGACTGAAAATTTGTTTTCAATAAAAATATGATATAAACTTGGCTGTTCAAGAGTTCAAAAAGATCAGGTTTGGAGCAAAGGACATAGTTTAGTGGTGCAAATGATAGAAGTGAGGTAGTCTCAGTTGAGTTGACAATCGCAATGTGAGGATTCGATTTTTACTTCTTACAAAATTTAAAATTGGACTCTTTGTGAATATCAATATAATAACTCTTGTATTCTAGCTTGTTGAAACACATCCCACAATTTATTTTTTTCACAATGTTTTAGCGATGACGCAGTGAAAAATCATCAAGGTTGGAGTTAACTTTTTGAGAAAGTCTTCGTCTTTGCAATAATGCATGATGATAAACCCAAAAAAGAAAGAGATCGAGGATAAATAATCTTCTAGTACTATCCTCTGTCAAACCTGTCTCTGTCTCTGTCTCTGTCTCTGTCTCTGGTGGATTTTCTTTTCTTTGCCTTTTTTTTCGTTTTTCCATATTCTCTCTCACACACACGCACAAAGACACATTCATTTCTTTATTCCCTTTTCCAGGCATATTTTGACCTCTCATTTGAGACCAATAGTTTTTCTGGGTTTTTGCCAAATTTATAAGATATCCCTTTTTACCATATTACACCAATCTCGTTATCTTTGTTCATTTTCACAGCATTTATTGGGCAATTTTTCTCAATATTTGTTTTATAGGTCAGTCCTGTTTCCTTCTTTATTTCATCTTAATCTTACTCAGAGAATCTTTGTTTTGATTCATACCTTTGATTCACTTCTCTTTTGATATTTGCTTTACAATTAGGTGTAGAA

**Document S5.** The CDS of *OfWRKY17* gene.

ATGATGGCGTTTGGTAACATGAACGAGCAAGCGGCGTTGCAGGAGGCGGCGACGGCGGGGTTGAAATCCATGGACCATTTAATCCGGCTGGTTTCTCACCAGGAACAACAAAACCCGCAAATCGACTGTACAGAAATCACTGATTTTACTGTTTCTAATTTTAAAAAGGTTATTTCTATTCTAAACCGGTCTGGCCATGCCCGATTCCGCCGTGCTCCGGTTCAAACTCCAGCACCAGAGCGGTTCACTTTCTTTGAAAATCATAATTTTGCTTCTGGGTCTAGTCAGATGCCTCCAGTCTCCGGGTTTAATAAGCCTCAGCCTCAGCCTCGGGATCTTAATCTCAATCTTTTCCCTGCAACTCCGGCGTCTGCGGTGGTGCCGGAACCAGCATTTACCCTTGATTTCGCGAAGCCAAGCGTGGTGGGTTCCACGCCTTTTGTTAAAGAGATTAGTAATGACGTGATGTGCAAGGACGGTTTCAGCTTATCGGCGGCGATGTCAACGTCAGGAAACTCGTCGACGACATTCGTTTCGTCTATAACCGGCGACGGGAGCGTGTCAAACGGCAAAGGTGGGCCGTCCTCTATGTTTATGGCTCCGGCAGGCCCCGCTATCTCCGCCGGAAAGCCTCCACTCTCCGGAAAGAGGTGCCGGGAGCATACCCAGTCAGATAACGTCTCCGGCCGGTGTCATTGCAAGAAGAGGAAATCTAAGGTGAAGAGGACAATTAGAGTCCCAGCCATTAGCTCAAAAATCGCGGATATTCCCGTTGACGAGTACTCATGGAGAAAGTATGGTCAAAAGCCGATCAAGGGCTCGCCATACCCACGGGGTTACTACAAGTGCAGTACCGTAAGGGGATGCCCGGCAAGAAAGCATGTGGAGAGGGCTACAGATGATCCATCGATGTTGATCGTCACTTATGAAGGGGAGCATAGGCATTCGCAACATGCAATGCAGGAGACCACGGCTTCGGAGGGTGGTCAATTCGTCGTGTTCGAGTCGACATGA

**Document S6.** The promoter of *OfWRKY17* gene.

TAAGTGCGTAGGTGACATCCGCGTGGAGGAGAGATAGCTAGAATTCTATTGACCCATTTTTTGAGGTCAATGGATTACATTGTGTAGAAACTTAAATATTTTTTCGCAAAACATTTTTCACACATATATAGGGAAATGCAAATGGACCAAGTGGGTTAATGGGTTCAAGGCCCAAGATATGATTGTTAGATTAATTAGTCCAATACAATTGAATAATCGATGCCAAGTCGACATATGTGACACCCCTTCCGTCATTCGCATGTCACATAATTTATATATATAACATAATCAATATAAGAAACACAATGAAAGAAATCCATGTGATATATGTGTGAATAATTTTGTTAATCATGCTATGTCCCAAAACTTCTAGTAAAACTACTATTAGGATTTGAGGTAGGATTAATAGGATTTGTGTCAAGAAGCTAATGGTAGCACAAGCCCACCCAAAAAAAAAAAAAAAAAAAGGCTTCTGAATTTTAAAATTATAGCCCCCAACTTAAATTTCTAATTGCAAGCTTGCTTCATTTTGATCGTTGATTGCCAATGAAGGAGATGCATTGATGTCTACTGACGTCACATTCGTGAATTTTACGTAATACTTATAAAATCTTTCCAAATTCCTATTAAACCCAACAAAATTATCACCAATATATATTTTAAAAACAACTAGAAGATTTCTACTATGATTAATATTTAATTAATAAAACACAAAAGTTCCGAGTTAAACATGTGCTTGTTTTATGATTGTCATATACTTCCTAGGAAAAAGCTCCCTGTCTCGACTTAGGGACAGGGTAAAAACAAGGGGCACACCGGGATAGGGAGCACGTCTCTACTTCCTAACGTTATTATGTCAAATAGCTTCACGACTTAAACAATTATAAGTTACCACAACTCATTTTTAGATTGCCATAATCCCATTCAAAAATTTTGTAATGTTATCATGTTGATTAGTTTTAAGACCCTAACAAATTTAAAAGCAGATTTGGTAACATGTATATTAGATAACGCAACATAGTATTAACAAAGAAAAAAAAAAAAAAATTATCCTTTATATTATCGATTATGTTTTAACACAGCATACCAAGTCCCTATTTGAACCCTATTTTTGAGAAAAATATGTTTTTAATTGTGAATTTTTGGAGGACGAGTAATTAAAAAGTATAAATAATTTTTGAATTTACGTATAAAATTATTTTATAAAAGAATTTGTAATTAAAAATGAGTTTTTAATTAAATTTTTACCAACATATACGATATGCTCATAATTTTTTCAATAAATTAAAAACGAATTTGCTATTTAAAAAAAAAAAAAAAAAAGAGTAAGCTATTAATCATTTTTCCCCCTAATAATTTATACATAAATCTCATGTGAGATTGCCAAAATCCCAACCACAATAATTAATGTACGTTACAAAGCTAATTAATGTCTCAAGTTAGGCGCCAAAGGGTATCCTAATCCCCACTTTGTCTTTTTCTCACCAACTCTGTAGTCTCGCCAGCTTTCTCTTTTCAACTTCATGAGAAATTCACTTTCATCTATTCATTATTTCCAAATTTGGGCCCCTCAATTTTTCTTTACTCAAGCACCCCAATTTCACTGTTAGATTATTTTTGTACTTTATGCATTAAGAAATGTACATTGTGCTGGAAGAATAGAATTGGATACTGGCATTATCAATTCCTAATACTTCCGCAATTGGGCCCCACCCAACATACAAAGGCCTCTATTTGGTCTCATACCGAATGCATTGAAGTCTTCAAAGAAAATCTCAACCGTCCGATTAAACTCATCCAGTCTTCCGGTCACCCTCCCTCTTCTTTATATACCCCCGCAACCCACCCCAATACACCATTACAATTTTCTATCCTTTTGCACTAAGTTTTTGTTCTTGATTTGGTTTTTGGGTTTTGATTTGTGATGCGGGTTCAGTAAATCTTCAAATTTGATTGAGGAAGCGTTCCCCCCTCCTTTTTGAGGATAAACGGCGGTGGAA
