# Supplementary material for: OfWRKY17-OfC3H49 module responding to high ambient temperature delays flowering via inhibiting OfSOC1B expression in Osmanthus fragrans
Source: Hortic Res. 2024 Sep 24;12(1):uhae273. doi: 10.1093/hr/uhae273 (PMC11725642; doi:10.1093/hr/uhae273)
Supplement: Web_Material_uhae273 [file web_material_uhae273.zip › Supplemental Figures.pdf]

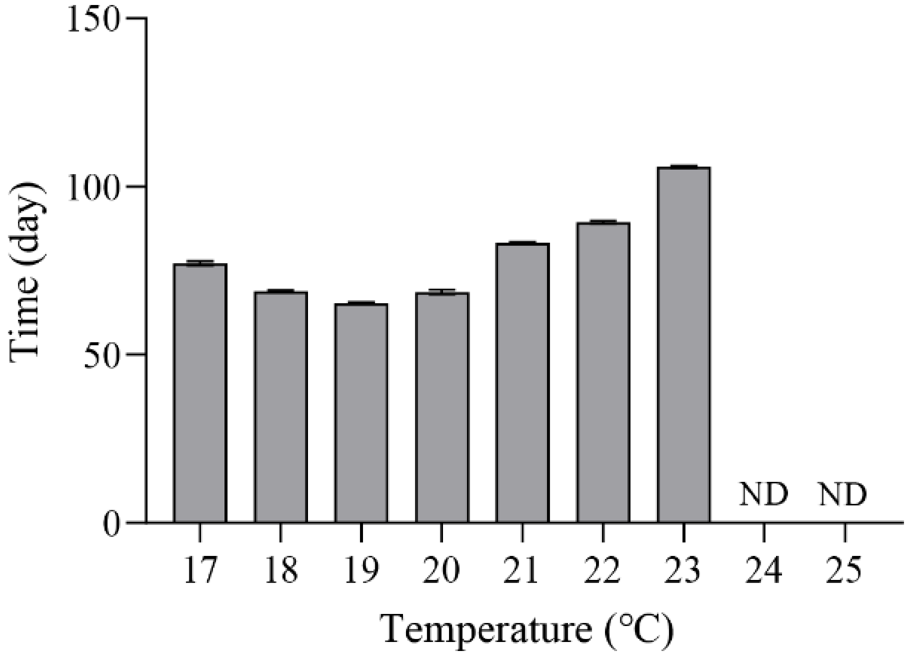

Fig S1 Flowering time of *O. fragrans* at different ambient temperatures.

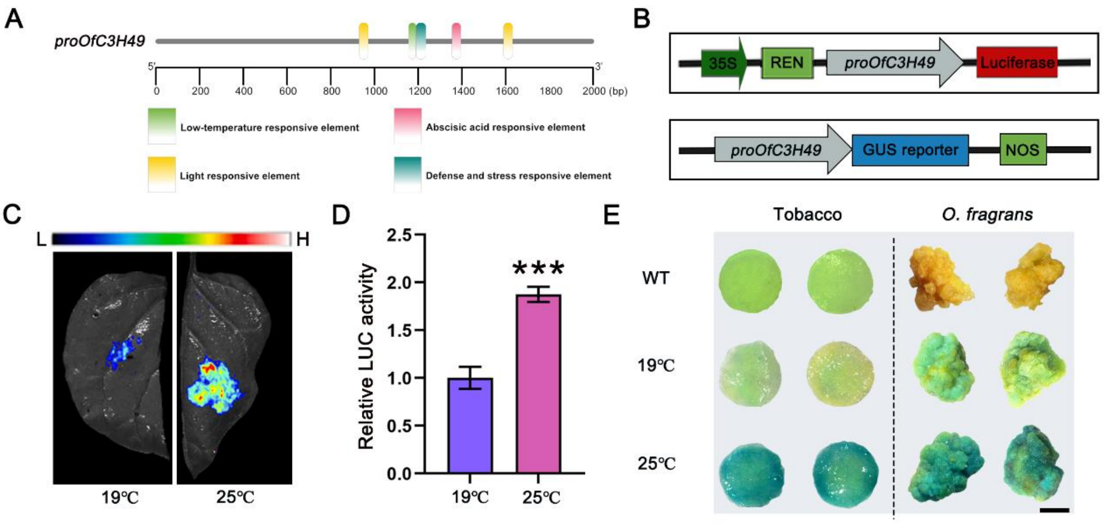

Figure S2. Analysis of *OfC3H49* promoter sequence and activity.

(A) The distribution of *cis*-regulatory elements (*CREs*) in the promoter of *OfC3H49*. **(B)** The vector structure of LUC and GUS reporter assays. **(C-D)** LUC activity assays driven by *OfC3H49* promoter in tobacco leaves at 19°C and 25°C. **(E)** GUS activity assays driven by *OfC3H49* promoter in tobacco leaves and *O. fragrans* calli at 19°C and 25°C. Data are presented as means ± SD (n = 3). *** represents *P*-value ≤ 0.001, based on Student's *t* test.

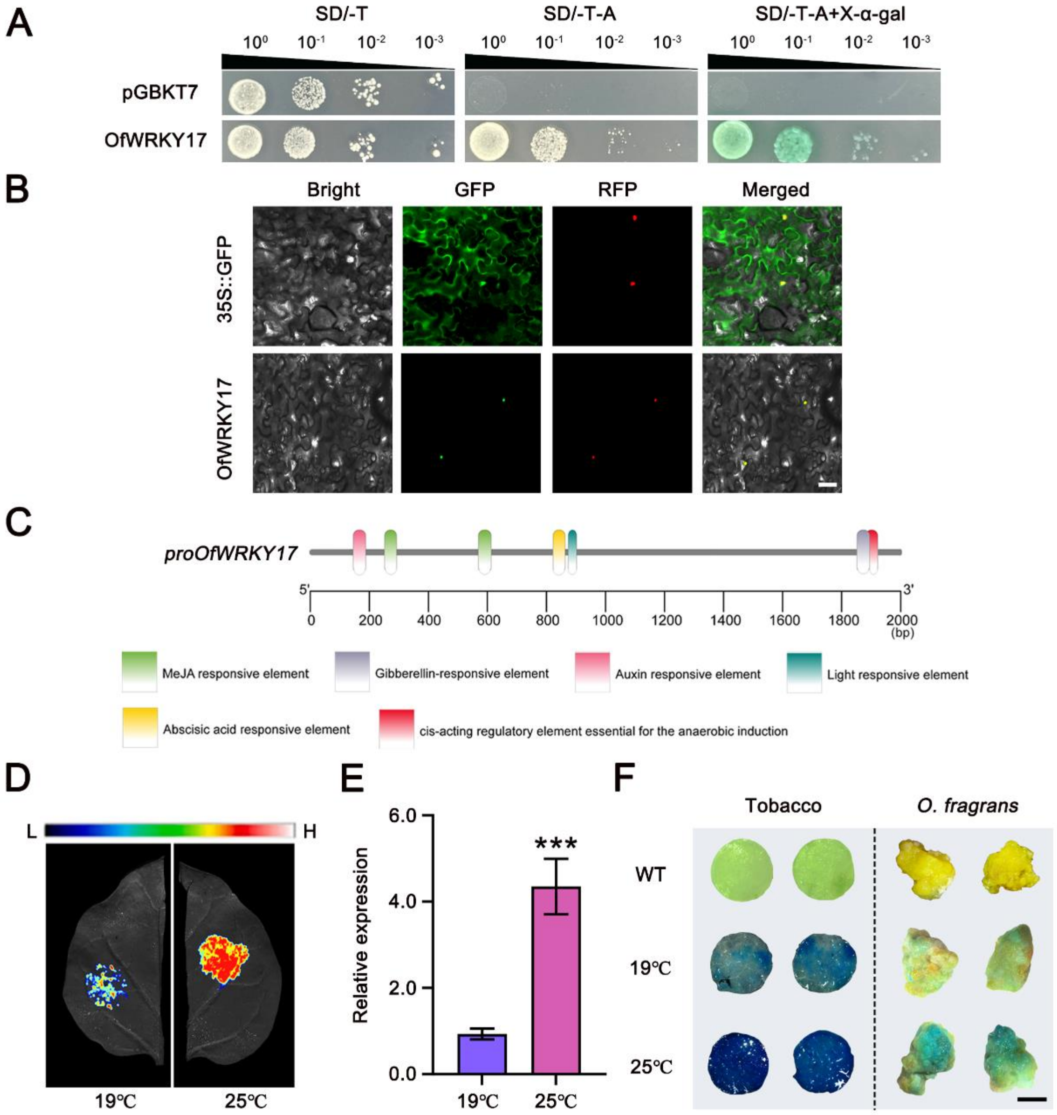

Figure S3. Analysis of OfWRKY17 protein characteristics and promoter activity. **(A)** Transcription activation assays of OfWRKY17 protein. **(B)** Subcellular location of OfWRKY17 protein. **(C)** The distribution of *CREs* in the promoter of *OfWRKY17*. **(D-E)** LUC activity assays driven by *OfWRKY17* promoter in tobacco leaves at 19°C and 25°C. **(F)** GUS activity assays driven by *OfWRKY17* promoter in tobacco leaves and *O. fragrans* calli at 19°C and 25°C. Data are presented as means ± SD (n = 3). *** represents *P*-value ≤ 0.001, based on Student's *t* test.

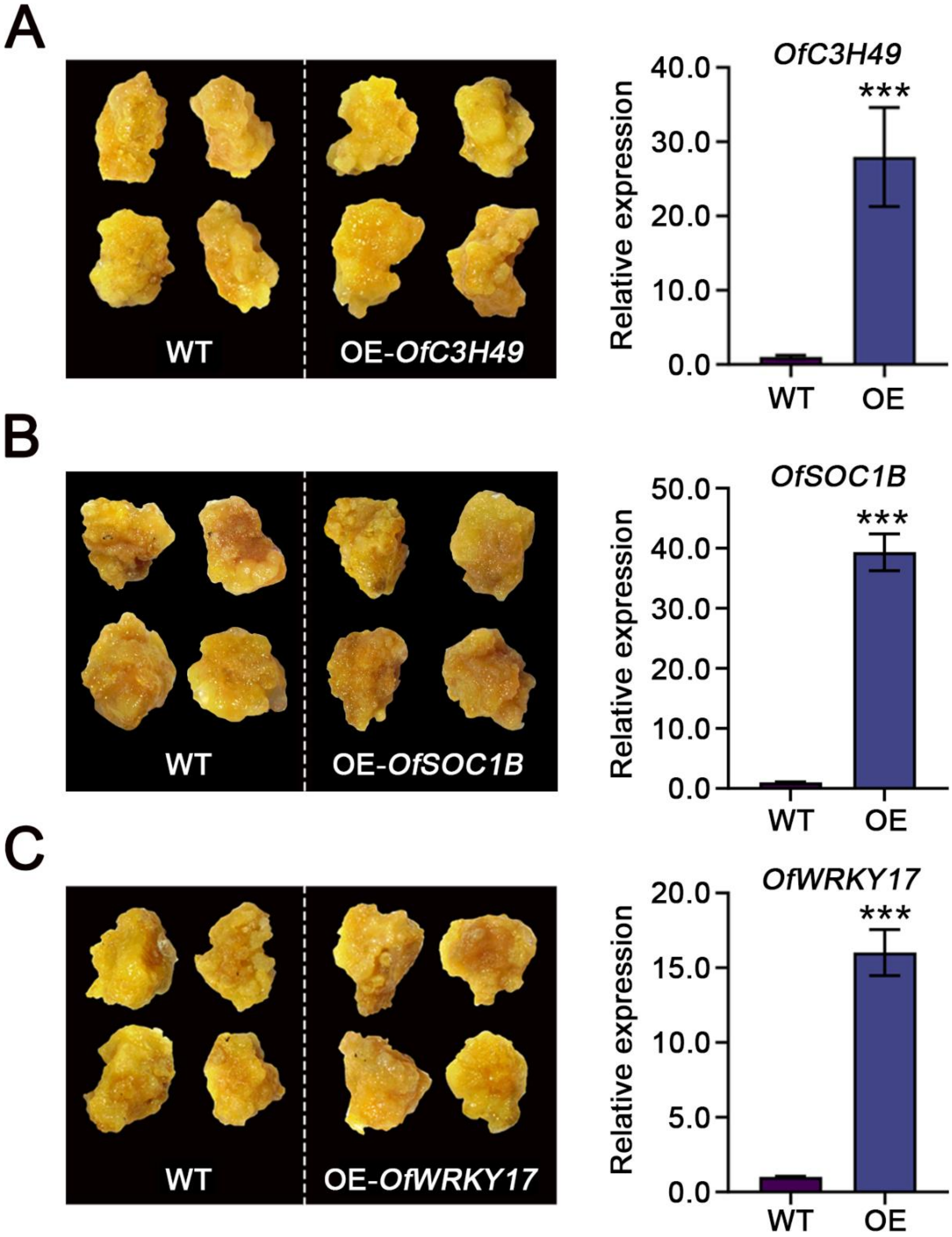

Figure S4. Validation of transgenic *O. fragrans* calli by qRT-PCR.
(A) qRT-PCR validation of *OfC3H49*-overexpressing *O. fragrans* calli. **(B)** qRT-PCR validation of *OfSOC1B*-overexpressing *O. fragrans* calli. **(C)** qRT-PCR validation of *OfWRKY17*-overexpressing *O. fragrans* calli. Data are presented as means ± SD (n = 3). *** represents P-value ≤ 0.001, based on Student's t test.

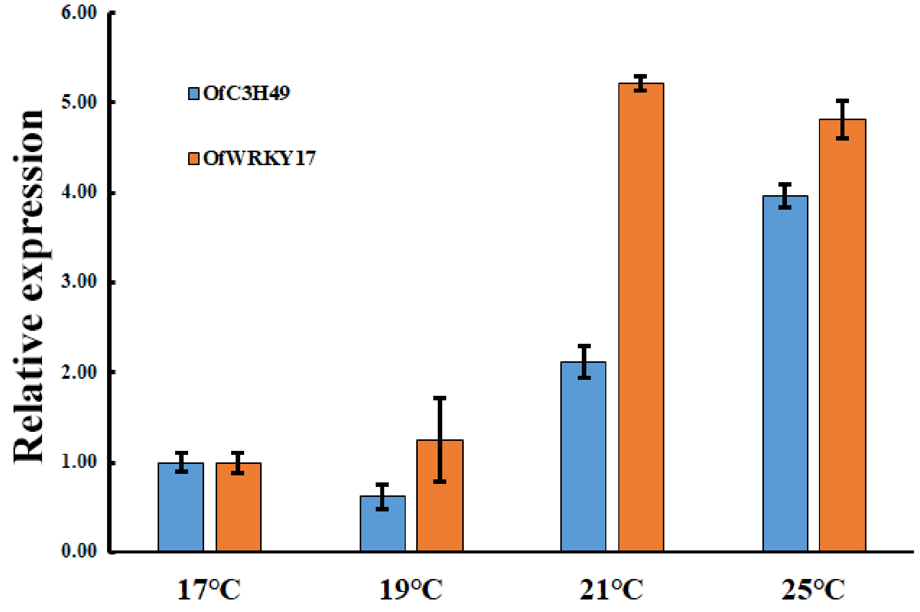

Figure S5 The expression of *OfC3H49* and *OfWRKY17* under 17°C, 19°C, 21°C, and 25°C condition
