# Supplementary material for: OfWRKY17-OfC3H49 module responding to high ambient temperature delays flowering via inhibiting OfSOC1B expression in Osmanthus fragrans
Source: Hortic Res. 2024 Sep 24;12(1):uhae273. doi: 10.1093/hr/uhae273 (PMC11725642; doi:10.1093/hr/uhae273)
Supplement: Web_Material_uhae273 [file web_material_uhae273.zip › Table S2.docx]

**Table S2.** **The primer sequences**

|  | **Gene name** | | **Sequences** | | | |  |  |
| --- | --- | --- | --- | --- | --- | --- | --- | --- |
| **qRT-PCR** | *AtACT* | | F: 5’-GGTAACATTGTGCTCAGTGGTGG-3’  R: 5’-AACGACCTTAATCTTCATGCTGC-3’ | | | |  |  |
|  | *AtSOC1* | | F: 5’-GTGTCAAATGTATTCGAGCAAG-3’  R: 5’-GAAGAACAAGGTAACCCAATGA-3’ | | | |  |  |
|  | *AtAP1* | | F: 5’-TCTTAAGCACATCCGCACT-3’  R: 5’-TCATTCCTCCTCATTGCCAT-3’ | | | |  |  |
|  | *AtFT* | | F: 5’-TGATATCCCTGCTACAACTGG-3’  R: 5’-CTATAGGCATCATCACCGTTC-3’ | | | |  |  |
|  | *AtLFY* | | F: 5’-ACGGCTTAGATTATCTGTTCCAC-3’  R: 5’-CTATATCCCAGCCATGACGAC-3’ | | | |  |  |
|  | *AtFLC* | | F: 5’-GTAAGCAGAGTTGTTGGAGACG-3’  R: 5’-TCTTGGCTTGTTTTGAACCT-3’ | | | |  |  |
|  | *AtTFL1* | | F: 5’-CCAAGCATAGGGATACATAGGTT-3’  R: 5’-GACAGGGAGACCAAGATCATACT-3’ | | | |  |  |
|  | *OfACT* | | F: 5’-CCCAAGGCAAACAGAGAAAAAAT-3’  R: 5’-ACCCCATCACCAGAATCAAGAA-3’ | | | |  |  |
|  | *OfSCO1B* | | F: 5’-GCTGAGGTTGCAGTTTTAAT-3’  R: 5’-TCGAGACGCTCTATGTTTTC-3’ | | | |  |  |
|  | *OfAP1a* | | F: 5’-GCAGAAGTGGCTTTGATTTG-3’  R: 5’-GTTTGCTGGTGACTGAGGTT-3’ | | | |  |  |
|  | *OfFT* | | F: 5’-CAAGCCCAAGTGATCCCAACCT-3’  R: 5’-GCCGGAATAACACGAAAACGAA-3’ | | | |  |  |
|  | *OfLFY* | | F: 5’-GGACTTGGGAGGTTTAGAGGACT-3’  R: 5’-CATTTCGTCAAGTTCCTCATCCT-3’ | | | |  |  |
|  | *OfWRKY17* | | F: 5’-GAAAGCATGTGGAGAGGGCT-3’  R: 5’-CGAATTGACCACCCTCCGAA-3’ | | | |  |  |
|  | *OfC3H3* | | F: 5’-ACTGTTTAGCCGTCGGTCAG-3’  R: 5’-CATCGACGTTGTTGTACGGC -3’ | | | |  |  |
|  | *OfC3H6* | | F: 5’-TACTGGATTGCTGCCTCTGC-3’  R: 5’-TCCAAACTTACACGACCCCG-3’ | | | |  |  |
|  | *OfC3H15* | | F: 5’-TGCCCGAAACCCAGTAGTTC-3’  R: 5’-CACCAAACTTGCACCCTTCG-3’ | | | |  |  |
|  | *OfC3H22* | | F: 5’-TGCCCGAAACCCAGTAGTTC-3’  R: 5’-CACCAAACTTGCACCCTTCG-3’ | | | |  |  |
|  | *OfC3H23* | | F: 5’-TCTTTGCTCACTCGACGGAC-3’  R: 5’-CAATCGGTGGAGAACCCGAA-3’ | | | |  |  |
|  | *OfC3H28* | | F: 5’-AGATGTTCCGAACCCGAACC-3’  R: 5’-CGAGATCCACTCCACATCCG-3’ | | | |  |  |
|  | *OfC3H48* | | F: 5’-GACTCTGGCATGGAAGTGCT-3’  R: 5’-CTTGCCACATGGCGTCTTTC-3’ | | | |  |  |
|  | *OfC3H49* | | F: 5’-GCTGCAGCTTTGAGCCTTTT-3’  R: 5’-TCCCATTTGCAGTGGGAGAC-3’ | | | |  |  |
|  | *OfC3H55* | | F: 5’-TTGCTCGACCTTGCTGCTAA-3’  R: 5’-CGTACCACGGTCCAACTTCA-3’ | | | |  |  |
| **Gene isolation** | *OfC3H49*–F | | 5’-ATGTGCAGTGGCCCAGAGC-3’ | | | |  |  |
|  | *OfC3H49–*R | | 5’-TCACCGGATGGGGCAGCA-3’ | | | |  |  |
|  | *proOfC3H49*–F | | 5’-TCAACAACATACTAACA-3’ | | | |  |  |
|  | *proOfC3H49–*R | | 5’-TACTTAAATCAGCTGCAGTCA-3’ | | | |  |  |
|  | *OfWRKY17*–F | | 5’-ATGATGGCGTTTGGTAACATGA-3’ | | | |  |  |
|  | *OfWRKY17–*R | | 5’-TCATGTCGACTCGAACACGACG-3’ | | | |  |  |
|  | *proOfWRKY17*–F | | 5’-TAAGTGCGTAGGTGACATCCGC-3’ | | | |  |  |
|  | *proOfWRKY17–*R | | 5’-TTCCACCGCCGTTTATCCT-3’ | | | |  |  |
| **EMSA probes** | *proOfC3H49*–biotin–F | | 5’-GCATATATCTAATTGACTTGAAATAAATGAA-3’ | | | |  |  |
|  | *proOfC3H49–*R | | 5’-TTCATTTATTTCAAGTCAATTAGATATATGC-3’ | | | |  |  |
|  | *proOfSOC1B*–biotin–F | | 5’-AGCATTCTCACAAAAAGTAATTTTCACTATA-3’ | | | |  |  |
|  | *proOfSOC1B–*R | | 5’-TATAGTGAAAATTACTTTTTGTGAGAATGCT-3’ | | | |  |  |
|  | | **Vectors** | **Plasmid name** | | **Primer sequences** | | | |
| **Vector constrction** | | pORE-R4-35SAA | | 35S::OfC3H49-GFP | | F: 5’-TTGGGGCCCAACGTTCTCGAG  ATGTGCAGTGGCCCAGAGC-3’  R: 5’-CGAATTCTCCCGGGTGGATCCAA  CCGGATGGGGCAGCACCA-3’ | |  |
|  |  | pORE-R4-35SAA | | 35S::OfWRKY17-GFP | | F: 5’-TTGGGGCCCAACGTTCTCGAG  ATGATGGCGTTTGGTAACATGA-3’  R: 5’-CGAATTCTCCCGGGTGGATCCAA  TCATGTCGACTCGAACACGACG-3’ | |  |
|  |  | pGreenII0800-LUC | | *ProOfC3H49*-LUC | | F: 5’-GGTACCGGGCCCCCCCTCGAG  CAACAACATACTAACATCGTTATTTATGC-3’  R: 5’-CGCTCTAGAACTAGTGGATCC  ACTTAAATCAGCTGCAGTCAATCAA-3’ | |  |
|  |  | pGreenII0800-LUC | | *proOfWRKY17*-LUC | | F: 5’-GGTACCGGGCCCCCCCTCGAG  TAAGTGCGTAGGTGACATCCGC-3’  R: 5’-CGCTCTAGAACTAGTGGATCC  TTCCACCGCCGTTTATCCT-3’ | |  |
|  |  | pGreenII0800-LUC | | *proOfSOC1B*-LUC | | F: 5’-GGTACCGGGCCCCCCCTCGAG  ATTTGTCATTTGTATCCTTCTCTACTTCG-3’  R: 5’-CGCTCTAGAACTAGTGGATCC  TTCTACACCTAATTGTAAAGCAAATATCA-3’ | |  |
|  |  | pCAMBIA 1300-GUS | | *ProOfC3H49*-GUS | | F: 5’-ACGACGGCCAGTGCCAAGCTT  CAACAACATACTAACATCGTTATTTATGC-3’  R: 5’-GGACTGACCACCCGGGGATCC  ACTTAAATCAGCTGCAGTCAATCAA-3’ | |  |
|  |  | pCAMBIA 1300-GUS | | *proOfWRKY17*-GUS | | F: 5’-ACGACGGCCAGTGCCAAGCTT  TAAGTGCGTAGGTGACATCCGC-3’  R: 5’-GGACTGACCACCCGGGGATCC  TTCCACCGCCGTTTATCCT-3’ | |  |
|  |  | pGADT7-AD | | pGADT7-OfC3H49 | | F: 5’-GCCATGGAGGCCAGTGAATTC  ATGTGCAGTGGCCCAGAGC-3’  R: 5’-CAGCTCGAGCTCGATGGATCC  TCACCGGATGGGGCAGCA-3’ | |  |
|  |  | pGADT7-AD | | pGADT7-OfWRKY17 | | F: 5’-GCCATGGAGGCCAGTGAATTC  ATGATGGCGTTTGGTAACATGA-3’  R: 5’-CAGCTCGAGCTCGATGGATCC  TCATGTCGACTCGAACACGACG-3’ | |  |
|  |  | pGBKT7-BD | | pGBKT7-OfC3H49 | | F: 5’-ATGGCCATGGAGGCCGAATTC  ATGTGCAGTGGCCCAGAGC-3’  R: 5’-CCGCTGCAGGTCGACGGATCC  TCACCGGATGGGGCAGCA-3’ | |  |
|  |  | pGBKT7-BD | | pGBKT7-OfWRKY17 | | F: 5’-ATGGCCATGGAGGCCGAATTC  ATGATGGCGTTTGGTAACATGA-3’  R: 5’-CCGCTGCAGGTCGACGGATCC  TCATGTCGACTCGAACACGACG-3’ | |  |
|  |  | pHis2 | | pHis2-*proOfC3H49* | | F: 5’-GACTCACTATAGGGCGAATTC  CAACAACATACTAACATCGTTATTTATGC-3’  R: 5’-CCAGGAATTTCTAGACCGCGG  ACTTAAATCAGCTGCAGTCAATCAA-3’ | |  |
|  |  | pHis2 | | pHis2-*proOfSOC1B* | | F: 5’-GACTCACTATAGGGCGAATTC  ATTTGTCATTTGTATCCTTCTCTACTTCG-3’  R: 5’-CCAGGAATTTCTAGACCGCGG  TTCTACACCTAATTGTAAAGCAAATATCA-3’ | |  |
|  |  | pGreenII-62sk-GAL4 | | GAL4-OfC3H49 | | F: 5’-CGCTCTAGAACTAGTGGATCC  ATGTGCAGTGGCCCAGAGC-3’  R: 5’-GGTACCGGGCCCCCCCTCGAG  CCGGATGGGGCAGCACCA -3’ | |  |
